# Association of serum uric acid with all-cause and cardiovascular mortality in obstructive sleep apnea

**DOI:** 10.1038/s41598-023-45508-2

**Published:** 2023-11-10

**Authors:** Zhe Yang, Tian Lv, Xiaoheng Lv, Fangyuan Wan, Hong Zhou, Xiaoling Wang, Lisan Zhang

**Affiliations:** 1 Nursing Department, Sir Run Run Shaw Hospital, Zhejiang University School of Medicine, Hangzhou, Zhejiang China; 2 Department of Neurology, Zhuji People’s Hospital , Zhuji, Zhejiang China; 3 Department of Neurology, Sir Run Run Shaw Hospital, Zhejiang University School of Medicine, Hangzhou, Zhejiang China

**Keywords:** Neurology, Neurological disorders, Sleep disorders

## Abstract

The study investigated the association between Serum Uric Acid (SUA) levels and all-cause as well as cardiovascular mortality in patients with Obstructive Sleep Apnea (OSA). This prospective cohort study enrolled participants with OSA from four cycles of the National Health and Nutrition Examination Survey (NHANES) conducted between 2005 and 2008, and 2015–2018. A weighted Cox proportional hazards model was used to assess adjusted hazard ratios (aHRs) and their corresponding 95% confidence intervals (CI) for all-cause and cardiovascular mortality. Additionally, multivariable logistic regression and restricted cubic splines (RCS) models were employed to examine nonlinear relationships between SUA and all-cause and cardiovascular mortality. Among the 5,584 OSA participants included in the study, covering the four NHANES cycles and with a median follow-up of 4.333 years, a total of 537 deaths were observed, including 108 deaths attributed to cardiovascular disease. Comparing the fourth quartile (Q4) of uric acid levels, both the fifth quartile (Q5) (aHRs = 1.51, 95% CI [1.08, 2.12]) and the second quartile (Q2) (aHRs = 1.53, 95% CI [1.04, 2.25]) of uric acid levels were independently associated with an increased risk of all-cause mortality. Furthermore, comparing the fourth quartile (Q4) of uric acid levels, the second quartile (Q2) (aHRs = 2.40, 95% CI [1.08, 5.35]) of uric acid levels were independently associated with an increased risk of cardiovascular mortality. The RCS model demonstrated a U-shaped pattern in the association between SUA and all-cause mortality in OSA, with an inflection point observed at 5.83 mg/dl. The findings of this study suggest a U-shaped association between serum SUA levels and all-cause mortality and nonlinearity association between serum SUA levels and all-cause mortality. Further studies are warranted to determine the causal relationship between SUA levels and all-cause and cardiovascular mortality.

## Introduction

Obstructive sleep apnea (OSA) is a symptomatic, recurrent obstruction of the upper airway that occurs during sleep, resulting in reduced airflow or complete cessation^1^. Globally, 936 million people suffer from OSA, with 425 million experiencing moderate to severe OSA^2^. The incidence of mild OSA in the general adult population ranges from 9 to 38%, reaching as high as 84% in certain elderly populations. Severe OSA affects 6% to 17% of the total adult population and is more prevalent in the elderly group (36%)^3^.

Serum uric acid (SUA) is a degradation product of purine. Intriguingly, experiments have demonstrated the beneficial antioxidant properties of uric acid^4^. In epidemiological studies, elevated SUA levels are widely acknowledged as a risk factor for both macrovascular and microvascular complications^5^.

Patients with obstructive sleep apnea (OSA) endure chronic intermittent hypoxemia and metabolic disruptions resulting from upper airway obstruction, hypopnea, and apnea during sleep. This leads to increased serum uric acid (SUA) levels^6^. Hyperuricemia is frequently observed in patients with OSA^7^. The severity of OSA correlates positively with SUA levels^8^, which may explain the heightened vulnerability of OSA patients to cardiovascular complications^9–12^. Cardiovascular disease (CVD) stands as the predominant global cause of mortality^13^. Hyperuricemia often coexists with other cardiovascular risk factors, including dyslipidemia, obesity, kidney disease, and uncontrolled glucose regulation. Nevertheless, recent research has proposed that low SUA concentrations also elevate the risk of mortality^5, 14–19^, although the underlying pathophysiological mechanisms remain unclear. Existing studies on uric acid have predominantly concentrated on specific diseases, such as cardiovascular disease^20^, diabetes mellitus (DM)^21^, the hemodialysis population, and liver disease.

The connection between uric acid and the risk of mortality in the OSA population remains underexplored. Thus, investigating the relationship between UA levels and OSA may yield novel insights into the effective management of these conditions.

## Material and methods

### Study population

The National Health and Nutrition Examination Survey (NHANES) is a comprehensive series of studies designed to evaluate the health and nutritional status of both adults and children in the United States (U.S.). Conducted by the Centers for Disease Control and Prevention (CDC), this survey is based on a carefully selected representative sample of the U.S. population. The NHANES database comprises five major components, encompassing demographic data, dietary information, physical examination results, laboratory findings, and questionnaire responses. The survey involves in-depth interviews and thorough physical examinations to gather relevant information. The collected data plays a crucial role in shaping public health policies and programs, evaluating the efficacy of health and nutrition initiatives, and tracking the trends of various diseases and conditions. All NHANES protocols were duly approved by the National Center for Health Statistics’ Research Ethics Review Board, and all participants provided informed consent before their involvement in the study. Moreover, all research conducted was in strict adherence to relevant guidelines and regulations. For comprehensive details on NHANES data collection, interested readers can access published information available at https://www.cdc.gov/nchs/nhanes.htm.

**Figure 1:**
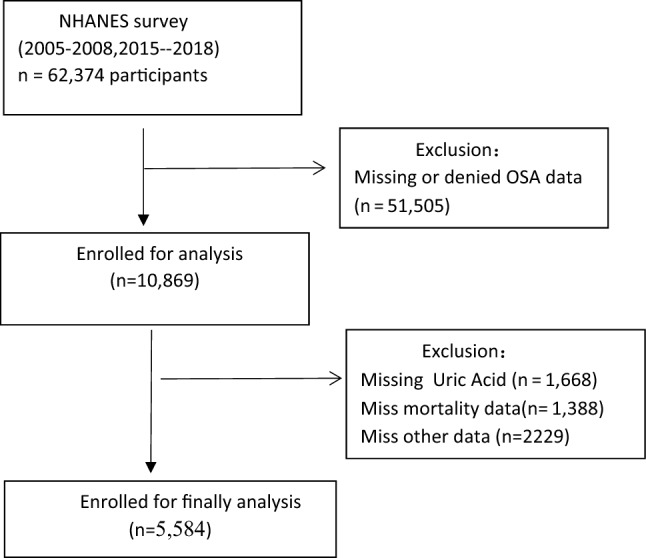
Flow chart of the study population.

For this study, we included a total of 62,374 participants from four cycles of the NHANES survey (2005–2006, 2007–2008, 2015–2016, 2017–2018). Participants who denied having OSA (n = 13,209) and those with missing OSA data (n = 38,296), SUA data (n = 1,668), mortality data (n = 1,388), stroke data (n = 1,273), chronic kidney disease(CKD) data (n = 228), DM data (n = 465), body mass index(BMI) data (n = 216), and eGFR (Estimated glomerular filtrate rate) data (n = 1) , hemoglobin(HB) data(n = 21), neutrophils data(n = 12), serum iron(n = 1) were excluded from the analysis. Therefore, a total of 5,584 participants were included in our analysis. Figure 1 presents the flow chart for the selection of the participants.

### Variables

The exposure variable in this study was SUA, while the outcome variables were all-cause mortality and cardiovascular mortality. These mortality data were collected from NHANES-linked National Death Index public access files through December 31, 2019.

OSA is ascertained through the responses to three dichotomous questions. These questions are as follows: (1) Do you snore for 3 or more nights per week? (2) Do you experience snorting, gasping, or stopping breathing for 3 or more nights per week? (3) Do you feel excessively sleepy during the day, ranging from 16 to 30 times per month, despite getting around 7 or more hours of sleep per night on weekdays or work nights? If individuals respond positively to any of these three questions, they are considered to exhibit symptoms indicative of OSA^23^.

Covariates were classified as demographics and medical conditions. Demographic information, including age, sex, marital status, education, and ethnicity, as well as medical conditions such as hypertension (defined as a history of physician-diagnosed hypertension, a measured average systolic blood pressure ≥ 140 mmHg, diastolic blood pressure ≥ 90 mmHg, or current use of antihypertensive medication), and DM (defined as a history of physician-diagnosed DM, hemoglobin A1c level ≥ 6.5%, fasting glucose (mmol/l) ≥ 7.0, random blood glucose (mmol/l) ≥ 11.1, two-hour oral glucose tolerance tests(OGTT)blood glucose (mmol/l) ≥ 11.1, or current use of DM medication or insulin) were collected through household interviews. Obesity (characterized by standard body index: under 25 kg/m^2^, 25–30 kg/m^2^, ≥ 30 kg/m^2^), cardiovascular disease (CVD), stroke, CKD, cancer, and hyperlipidemia,diuretics drug were also included in the analysis.

### Statistical analysis

The statistical analyses were performed using R Studio 4.2.0. The level of statistical significance was set at *p* < 0.05. Complex sampling designs and sampling weights in accordance with the NHANES analytic guidelines were considered in our study. All analyses were performed using mobile examination centers (MECs) weights. Baseline characteristics are presented as means and standard errors (SEs) for continuous variables, and proportions for categorical variables. SUA concentrations were categorized into quintiles. The quintiles of SUA groups were compared using Student's t-test for continuous variables and chi-square test for categorical variables. Cox proportional hazards models were used to estimate adjusted hazard ratios (aHRs) and 95% confidence intervals (CI) for the association between SUA concentrations and all-cause, CVD mortality. Three models were built to provide statistical inference. Model 1 included only SUA concentrations. Model 2 included Model 1, sex, and age. Model 3 included Model 2, sex, education status, eGFR, BMI, ethnicity, smoking status, history of hypertension, DM, CKD, CVD, stroke, cancer, diuretics drug,and hyperlipidemia. Stratified analyses by sex, race, BMI, age, CKD, hyperlipidemia, DM, hypertension, education, CVD, stroke, and smoking status were performed. Restricted cubic spline (RCS) regression was used to flexibly model the association of SUA with all-cause mortality and cardiovascular mortality, with the reference value being the inflection point of SUA.

## Results

### Basic characteristics

This study utilized a dataset comprising 5,584 patients with OSA, which is considered representative of a population-based sample size of 61,957,202 participants. The median follow-up duration was 4.333 years (interquartile range [IQR], 2.604–12.083). Table 1 presents the basic characteristics of the 5,584 subjects. Among the total sample, 3,020 (54.08%) were male, and there were 531 (9.51%) recorded cases of mortality. SUA levels were categorized into quintiles: Q1 (< 4.4 mg/dl), Q2 (4.4–5.2 mg/dl), Q3 (5.2–5.899 mg/dl), Q4 (5.9–6.79 mg/dl), and Q5 (≥ 6.8 mg/dl). Table 1 revealed significant differences between SUA quintiles and various variables, including BMI, sex, CKD, DM, hypertension, HB, bilirubin, red cell distribution width (RDW), platelet, serum iron, and hyperlipidemia, used diuretics in participants with higher SUA (all *p* < 0.0001). Additionally, higher SUA levels were significantly associated with lower eGFR at baseline (*p* < 0.001). Notably, there were significant differences in survival status across the SUA quartiles (*p* < 0.005). Participants with higher SUA levels exhibited negative significant differences in stroke, smoking status, education, ethnicity, CVD, chronic obstructive pulmonary disease (COPD), lymphocyte, neutrophils, cancer, and age. Survival time demonstrated a significant U-shaped relationship, with lower values observed in participants with SUA ≤ 4.4 mg/dl and ≥ 6.8 mg/dl (*p* < 0.01).

**Table 1:** Baseline characteristics of study participants based on the SUA quintiles. Mean ± SEs for continuous variables: *P* value was calculated by weighted Student’s t test. Number (%) for Categorical variables: *P* value was calculated by weighted chi-square test. Abbreviations: BMI, body mass index; CVD, cardiovascular disease; DM, diabetes mellitus; IFG, Impaired Fasting Glucose. IGT, Impaired Glucose Tolerance; CKD, chronic kidney disease; GFR, Glomerular filtration rate. RDW, Red cell distribution width. COPD, Chronic Obstructive Pulmonary Disease; HB, hemoglobin. * CKD-EPI, Chronic Kidney Disease Epidemiology Collaboration.

	Total	Q1	Q2	Q3	Q4	Q5	*P* value
Age(year)	48.967(0.366)	48.770(0.645)	48.874(0.720)	49.184(0.710)	49.265(0.575)	48.739(0.534)	0.92
Sex							< 0.0001*
Female	2564(45.917)	885(78.508)	607(57.522)	468(38.737)	358(31.179)	246(18.746)	
Male	3020(54.083)	282(21.492)	507(42.478)	650(61.263)	734(68.821)	847(81.254)	
Education							0.404
Uncollege	2704(48.424)	566(40.547)	576(43.968)	540(41.857)	505(38.925)	517(42.384)	
College	2880(51.576)	601(59.453)	538(56.032)	578(58.143)	587(61.075)	576(57.616)	
Race							0.334
White	2402(43.016)	468(67.344)	459(68.544)	479(68.584)	497(70.489)	499(70.160)	
Black	1175(21.042)	249(11.537)	217(10.316)	221(9.882)	225(9.774)	263(11.551)	
Other	2007(35.942)	450(21.119)	438(21.140)	418(21.535)	370(19.738)	331(18.289)	
BMI							< 0.0001*
< 25	967(17.317)	333(32.440)	240(23.284)	174(13.735)	144(12.276)	76(5.881)	
25–30	1739(31.143)	360(29.630)	352(29.683)	363(34.181)	338(30.933)	326(31.073)	
> = 30	2878(51.54)	474(37.930)	522(47.033)	581(52.084)	610(56.791)	691(63.046)	
Stroke							0.634
Yes	278(4.979)	53(3.768)	52(3.452)	35(3.120)	64(3.841)	74(4.503)	
CKD							< 0.0001*
Yes	1099(19.681)	173(11.632)	171(12.331)	199(14.350)	234(15.909)	322(22.669)	
DM							< 0.0001*
No	3697(66.207)	833(76.360)	772(75.301)	750(72.076)	687(66.827)	655(66.439)	
IGT	174(3.116)	32(2.633)	34(2.420)	34(3.566)	34(3.224)	40(3.589)	
IFG	347(6.214)	41(4.243)	53(4.470)	76(6.930)	90(10.657)	87(9.707)	
DM	1366(24.463)	261(16.764)	255(17.809)	258(17.428)	281(19.293)	311(20.264)	
Hypertension							< 0.0001*
Yes	2841(50.878)	496(39.919)	514(40.688)	548(45.695)	597(50.477)	686(55.911)	
CVD							0.119
Yes	789(14.13)	136(10.947)	151(10.265)	123(9.106)	173(11.023)	206(13.248)	
Cancer							0.088
Yes	564(10.1)	124(13.427)	120(10.926)	112(11.764)	101(8.612)	107(9.126)	
Smoke							0.217
Never	2725(48.8)	627(50.449)	569(52.232)	551(47.273)	488(46.207)	490(47.022)	
Former	1515(27.131)	260(24.586)	280(24.176)	306(27.511)	320(29.160)	349(30.219)	
Now	1344(24.069)	280(24.965)	265(23.592)	261(25.216)	284(24.633)	254(22.760)	
Hyperlipidemia							< 0.0001*
Yes	4268(76.433)	817(68.892)	820(73.643)	864(78.645)	872(80.924)	895(77.877)	
COPD							0.461
Yes	342(6.125)	69(5.919)	74(5.887)	58(4.100)	70(5.660)	71(4.889)	
Iron(ug/dl)	85.829(0.814)	78.475(1.421)	83.619(1.291)	86.239(1.572)	89.963(1.459)	91.223(1.433)	< 0.0001*
Lymphocyte%	29.976(0.174)	30.468(0.357)	30.307(0.309)	29.986(0.331)	29.785(0.286)	29.308(0.329)	0.085
Neutrophils%	58.405(0.193)	58.323(0.427)	58.360(0.356)	58.334(0.396)	58.268(0.321)	58.752(0.366)	0.802
HB	14.466(0.047)	13.767(0.066)	14.189(0.058)	14.613(0.074)	14.786(0.066)	15.010(0.091)	< 0.0001*
RDW	13.311(0.027)	13.460(0.046)	13.261(0.045)	13.281(0.059)	13.250(0.046)	13.298(0.050)	0.003*
Bilirubin	0.612(0.007)	0.496(0.011)	0.589(0.012)	0.631(0.013)	0.647(0.014)	0.703(0.016)	< 0.0001*
Platelet count (1000cells/ul)	257.132(1.418)	265.216(2.772)	257.785(2.947)	257.145(2.708)	256.104(2.564)	248.851(2.513)	< 0.001*
eGFR(CKD-EPI*)	93.139(0.541)	99.376(0.845)	94.257(1.019)	93.588(0.864)	90.553(0.786)	87.533(0.837)	< 0.0001
Drug of diuretic							< 0.0001
No	5018(91.683)	1082(94.221)	1046(95.408)	1010(91.653)	982(91.133)	898(85.866)	
Yes	566(8.317)	85(5.779)	68(4.592)	108(8.347)	110(8.867)	195(14.134)	
Survive status							0.004*
Alive	5053(90.491)	1077(94.110)	1011(92.774)	1024(94.006)	1001(94.023)	940(90.138)	
Dead	531(9.509)	90(5.890)	103(7.226)	94(5.994)	91(5.977)	153(9.862)	

### Associations of SUA with all-cause and CVD mortality in OSA

During the follow-up period of 5,584 participants, a total of 537 deaths occurred due to all causes, including 108 deaths related to CVD. Table 2 illustrates the relationship between SUA levels and both all-cause and CVD mortality in individuals with OSA after adjusting for various factors such as age, sex, race, education, BMI, eGFR, red cell distribution width (RDW), HB, lymphocyte count, neutrophil count, iron levels, bilirubin levels, platelet count, smoking status, history of hypertension, DM, CKD, chronic obstructive pulmonary disease (COPD), stroke, pre-existing CVD, cancer, used diuretics, and hyperlipidemia. The aHRs and corresponding 95% CI for different SUA categories (< 4.4, 4.4–5.19, 5.2–5.899, 5.9–6.799, and ≥ 6.8 mg/dl) were as follows: 1.33 (0.82, 2.15), 1.53 (1.04, 2.25), 1.18(0.79, 1.77), 1.00 (reference), and 1.51 (1.08, 2.12) for all-cause mortality (p trend = 0.03); and 1.12 (0.37, 3.42), 2.40(1.08, 5.35), 1.45 (0.62, 3.36), 1.00 (reference), and 1.77 (0.88, 3.56) for CVD mortality (*p* trend = 0.098).

**Table 2:** Multivariable Cox regression analyses demonstrating associations of SUA and all-cause mortality, CVD mortality. Model 1 only comprised SUA only. Model 2 included model1, age, sex. Model 3 included demographic variables (age, sex, race, education), BMI, GFR, RDW, HB, Lymphocyte, Neutrophils, iron, bilirubin, Platelet count, smoke, history of hypertension, DM, CKD, COPD, stroke, CVD, cancer, and hyperlipidemia, drug for diuretic. *P < 0.05.

	Event	*P*-value for log-rank test	Multivariable adjusted (HR, 95% CI)*		
			Model1	Model2	Model3
All-cause mortality		< 0.0001			
Q1	90		1.12(0.75,1.66)	1.29(0.88,1.91)	1.33(0.82,2.15)
Q2	103		1.21(0.87,1.672)	1.45(1.05,1.99)*	1.53(1.04,2.25)*
Q3	94		1.004(0.67,1.51)	1.109(0.78,1.58)	1.18(0.79,1.77)
Q4	91		1	1	1
Q5	153		1.76 (1.29,2.40) *	1.79(1.31,2.44) *	1.51(1.08,2.12)*
Ptrend			0.001	< 0.001	0.03
SUA	531		1.13(1.04,1.22)*	1.091(1.01,1.19)*	1.01(0.93,1.11)
CVD mortality		0.033			
Q1	16		0.80(0.374,1.716)	1.02(0.45,2.30)	1.12(0.370,3.42)
Q2	31		1.59(0.89,2.85)	2.10(1.10,3.99)*	2.40(1.08,5.35)*
Q3	20		1.14(0.53,2.47)	1.33(0.61,2.87)	1.45(0.62,3.36)
Q4	21		1	1	1
Q5	46		2.02(1.13,3.61)*	2.202(1.17,4.15)*	1.77(0.88,3.56)
Ptrend			0.011	0.014	0.07
SUA	134		1.214(1.07,1.38)	1.16(0.99,1.34)	1.05(0.89,1.24)

### Nonlinear association of SUA with all-cause and CVD mortality

**Figure 2:**
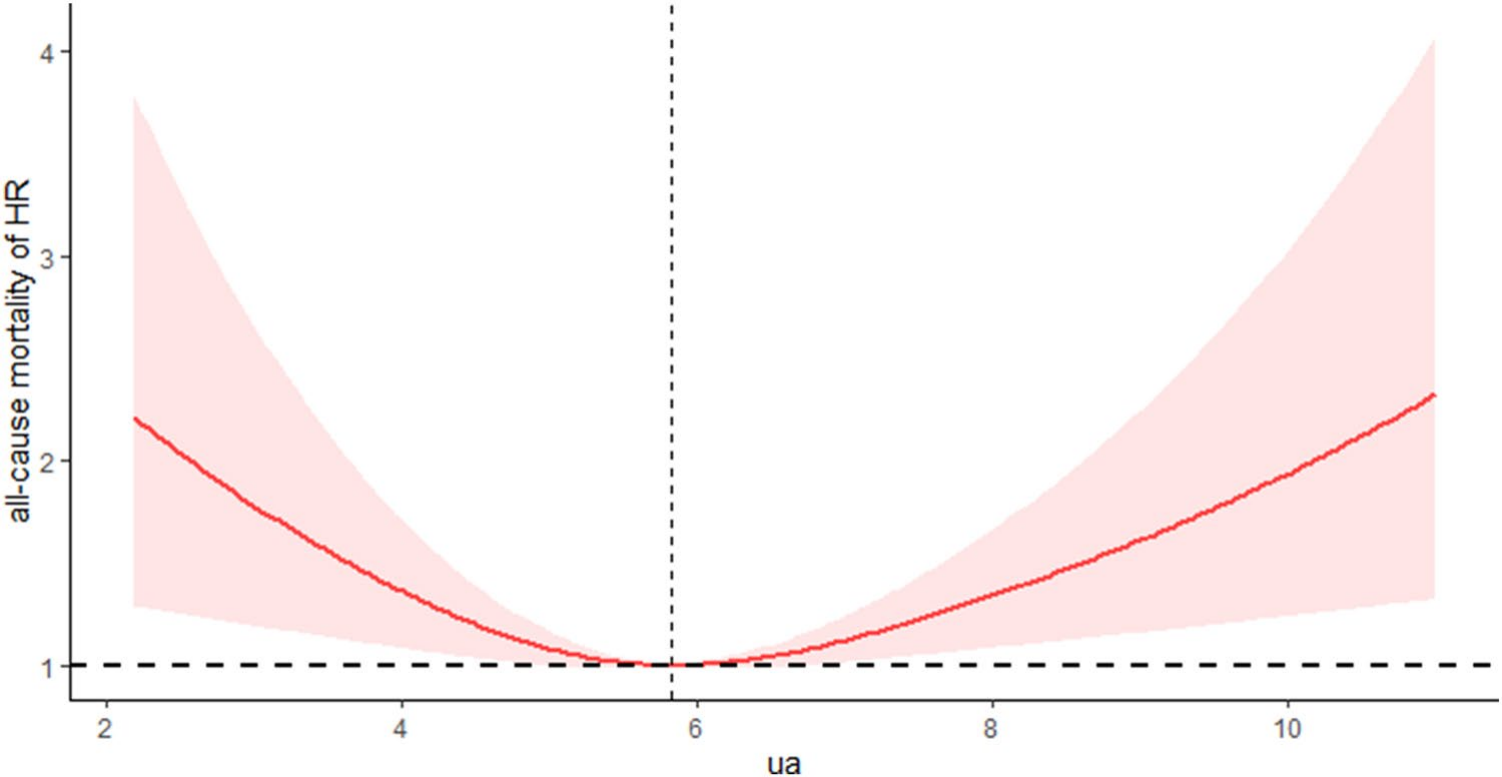
Multivariable-adjusted HRs for all-cause mortality by uric acid level.

**Figure 3:**
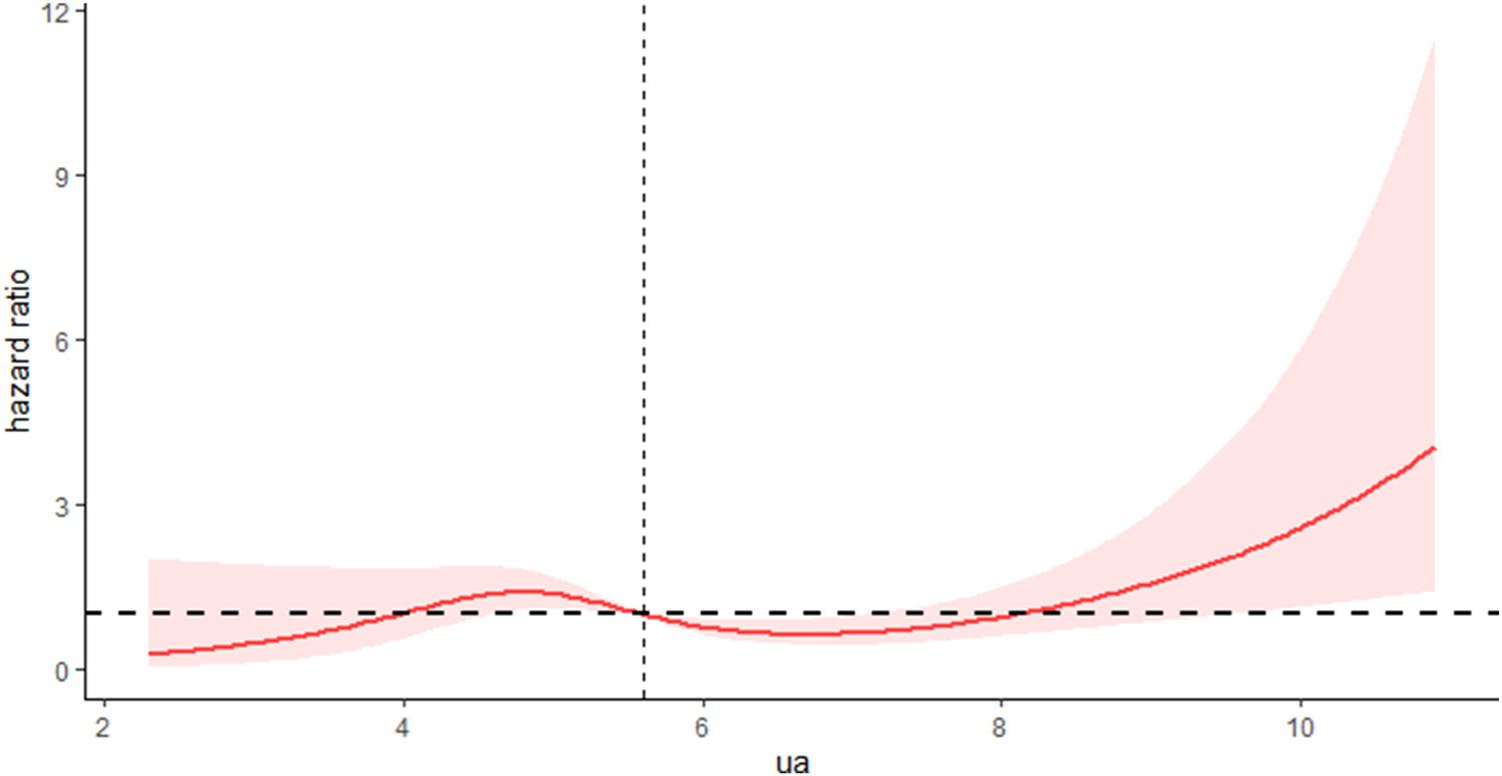
Multivariable-adjusted HRs for CVD mortality by uric acid level.

As shown in Table 2, after adjusting for age, sex, education status, BMI, ethnicity, smoking, eGFR, history of hypertension, DM, CKD, CVD, stroke, cancer, and hyperlipidemia, a U-shaped association was observed between SUA and all-cause mortality in OSA. The lowest aHRs were around the level of 5.83 mg/dl (*P* < 0.001 for nonlinearity). RCS models suggested that the inflection point for all-cause mortality (Fig. 2) was 5.83 mg/dl. On the left side of the inflection point, the aHRs for all-cause mortality were 0.87 (95% CI: 0.70, 1.07; *p* = 0.18) for every 1 mg/dl increase in SUA. On the right side of the inflection point, the aHRs for all-cause mortality were 1.29 (95% CI: 1.12, 1.49; *p* < 0.001). The RCS models revealed an inflection point for CVD mortality at 6.66 mg/dl (Fig. 3). On the left side of this inflection point, there was a significant inverse association between SUA levels and CVD mortality, with aHRs of 0.84 (95% CI: 0.72, 0.98; *p* = 0.032) for every 1 mg/dl increase in SUA. Conversely, on the right side of the inflection point, there was a positive association, with aHRs of 1.34 (95% CI: 1.14, 1.58; *p* < 0.001). All-cause and CVD mortality were observed before and after the inflection point in Table 3.

**Table 3:** The results of two-piecewise linear regression model between serum uric acid and all-cause, CVD mortality. Adjusted for the variables included demographic variables (age, sex, race, education), BMI, GFR, RDW, HB, LYM, NEU, iron, bilirubin, platelet count, smoke, history of hypertension, DM, CKD, COPD, stroke, CVD, cancer, and hyperlipidemia, drug of diuretic.

	Adjusted HR*	95% CI	*P*-value	*p* for interaction
All-cause mortality				
Inflection point(mg/dl)	5.83			0.001
SUA < 5.83	0.87	(0.70,1.07)	0.18	
SUA ≥ 5.83	1.29	(1.12,1.49)	< 0.001	
CVD mortality				< 0.001
Inflection point(mg/dl)	6.66			
SUA ≥ 6.66	1.34	(1.14,1.58)	< 0.001	
SUA < 6.66	0.84	(0.72,0.98)	0.032	

**Figure 4:**
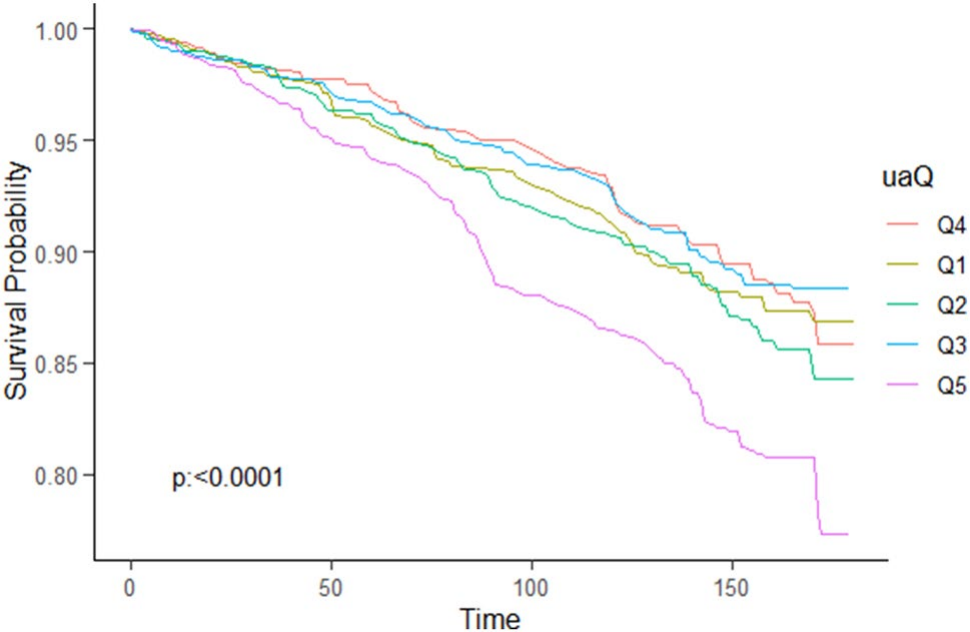
Kaplane-Meier survival analysis was used to evaluate between SUA and all-cause mortality. The p-value was obtained by log rank test. Log rank *P* < 0.0001.

**Figure 5:**
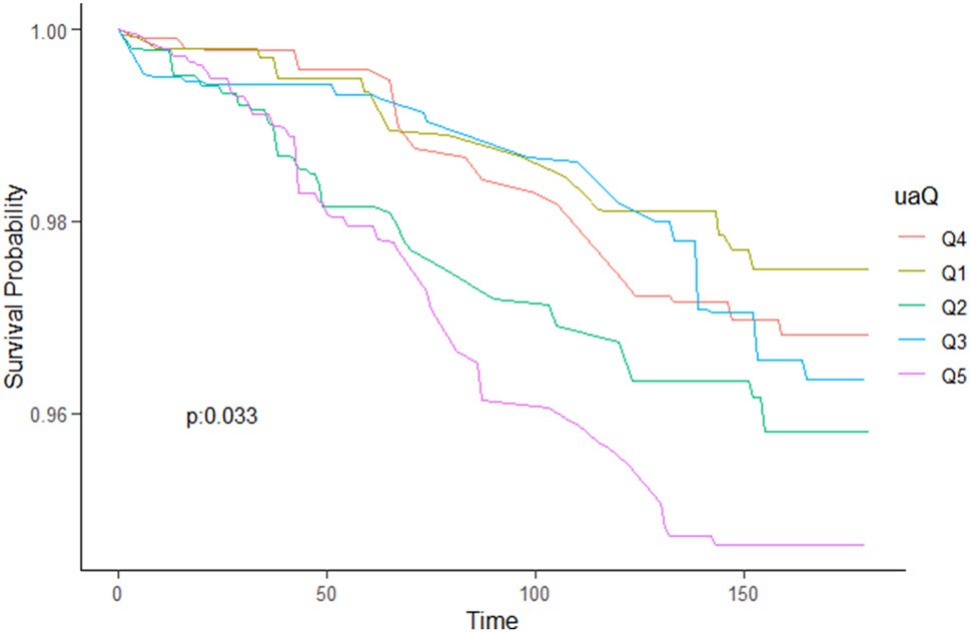
Kaplane-Meier survival analysis was used to evaluate between SUA and all-cause mortality. The p-value was obtained by log rank test. Log rank *P* = 0.033.

As depicted in the Kaplan–Meier survival curves, patients with OSA who had SUA levels of ≥ 6.8 mg/dl (Q5) exhibited significantly higher rates of all-cause mortality (Fig. 4) and CVD mortality (Fig. 5) during the follow-up period.

In the female group, our analysis revealed no association between SUA levels and all-cause mortality after adjusting for multiple variables (model 3), as shown in Table S1. Conversely, in the male group, we observed that both Q1 and Q5 SUA levels were associated with an increased risk of all-cause mortality after adjusting for multiple variables (model 3), as presented in Table S1.

Utilizing RCS models, we determined that the inflection point for all-cause mortality in females was 5.277 mg/dl (Fig. S1). Notably, there were no significant differences in female all-cause mortality before and after the inflection point, as demonstrated in Table S2. Conversely, for males, the RCS models indicated an inflection point of 6.219 mg/dl for all-cause mortality (Fig. S2). Substantial differences in male all-cause mortality were observed before and after the inflection point in Table S2.

In the elderly group, we observed that low (Q1, Q2) and high (Q5) SUA levels were associated with an increased risk of all-cause mortality after adjusting for multiple variables (model 3), as presented in Table S3. In contrast, in the younger group, there was no association between SUA levels and all-cause mortality after adjusting for multiple variables (model 3), as shown in Table S3. The RCS models indicated an inflection point of 6.32 mg/dl for all-cause mortality in the elderly (Fig. S3). On the left side of the inflection point, the aHRs for all-cause mortality were 0.74 (95% CI: 0.64, 0.86; *p* < 0.001) for every 1 mg/dl increase in SUA. On the right side of the inflection point, the aHRs for all-cause mortality were 1.20 (95% CI: 0.98, 1.48; *p* = 0.074). However, in the young group, a linear correlation was observed for all-cause mortality, as indicated by the RCS models (Fig. S4).

### Stratified analyses

Stratified analyses were conducted to assess the robustness of the relationship between SUA and all-cause mortality. In the adjusted model 3, the results of the stratified analysis demonstrated that the association between SUA and all-cause mortality remained consistent across most sub-populations, as presented in Table 4. Notably, a significant interaction was observed between SUA and the stratified variables, namely age and BMI. Specifically, our findings revealed a stronger association between SUA and all-cause mortality among elderly individuals (≥ 60 years old) with OSA, as indicated by a distinct interaction test. However, no significant interaction test was observed for male individuals with OSA.

**Table 4:** Stratification analysis of SUA with all-cause mortality in OSA. Adjusted for age, sex, education status, BMI, eGFR, race, smoke, history of hypertension, DM, CKD, CVD, stroke, cancer and Hyperlipidemia, drug of diuretic. *p < 0.05.

	Q4	Q1	*p*	Q2	*p*	Q3	*p*	Q5	*p*	*p* for trend(character2integer)	*p* for trend(Median value)	*p* for interaction
Age												0.021*
< 60	ref	0.934(0.302,2.887)	0.906	0.951(0.480,1.886)	0.887	0.688(0.333,1.421)	0.312	1.173(0.778,1.768)	0.446	0.918	0.529	
> = 60	ref	2.528(1.592,4.013)*	< 0.0001	2.245(1.477,3.414)*	< 0.001	1.581(1.108,2.255)*	0.012	1.675(1.030,2.725)*	0.038	0.122	0.006	
BMI												0.002*
25–30	ref	0.727(0.322,1.642)	0.443	2.031(0.960,4.294)	0.064	1.447(0.794,2.636)	0.227	1.846(0.932,3.659)	0.079	0.042	0.351	
> = 30	ref	2.850(1.457,5.575)*	0.002	1.199(0.668,2.153)	0.542	1.228(0.665,2.267)	0.511	1.531(0.931,2.518)	0.093	0.546	0.131	
< 25	ref	0.761(0.330,1.755)	0.522	1.275(0.610,2.666)	0.518	0.890(0.360,2.200)	0.801	1.680(0.841,3.354)	0.142	0.131	0.112	
CKD												0.998
Yes	ref	1.579(0.886,2.813)	0.121	1.645(0.967,2.800)	0.066	1.239(0.823,1.865)	0.304	1.497(0.973,2.304)	0.067	0.134	0.667	
No	ref	1.260(0.690,2.302)	0.452	1.624(0.986,2.676)	0.057	1.113(0.611,2.027)	0.727	1.678(1.107,2.545)	0.015	0.065	0.797	
Sex												0.083
Female	ref	1.268(0.666,2.415)	0.469	1.297(0.722,2.330)	0.384	1.290(0.678,2.453)	0.438	0.971(0.473,1.997)	0.937	0.869	0.278	
Male	ref	2.111(1.142,3.901)*	0.017	2.080(1.331,3.250)*	0.001	1.166(0.760,1.791)	0.482	1.948(1.347,2.818)*	< 0.001	0.006	0.631	
Hyperlipidemia												0.488
Yes	ref	1.552(0.912,2.641)	0.105	1.754(1.179,2.611)	0.006	1.312(0.837,2.056)	0.237	1.428(0.960,2.122)	0.078	0.141	0.225	
No	ref	1.697(0.722,3.987)	0.225	1.251(0.546,2.865)	0.596	0.957(0.402,2.278)	0.92	2.173(1.118,4.224)	0.022	0.176	0.593	
DM												0.45
DM	ref	1.596(0.677,3.760)	0.285	1.629(0.910,2.918)	0.101	1.253(0.645,2.434)	0.506	1.321(0.712,2.451)	0.377	0.476	0.362	
No	ref	1.389(0.712,2.706)	0.335	1.696(1.064,2.703)	0.026	1.007(0.495,2.049)	0.984	1.979(1.232,3.181)	0.005	0.048	0.342	
IFG	ref	3.067(0.833,11.292)	0.092	0.725(0.117, 4.499)	0.73	2.450(0.767, 7.819)	0.13	1.153(0.252, 5.277)	0.854	0.501	0.362	
IGT	ref	16.364(1.522,175.904)	0.021	15.734(0.985,251.406)	0.051	0.978(0.021, 45.039)	0.991	1.901(0.260, 13.896)	0.527	0.569	0.073	
Hypertension												0.318
Yes	ref	1.537(0.905,2.610)	0.111	1.654(1.105,2.477)*	0.014	1.282(0.797,2.061)	0.306	1.372(0.914,2.061)	0.127	0.207	0.156	
No	ref	2.068(0.862,4.964)	0.104	1.821(0.953,3.478)	0.07	1.135(0.496,2.596)	0.765	2.506(1.357,4.629)	0.003*	0.058	0.847	
Smoke												0.743
Former	ref	1.421(0.619,3.262)	0.408	1.735(0.997,3.021)	0.051	1.275(0.676,2.404)	0.453	1.388(0.753,2.559)	0.293	0.296	0.522	
Never	ref	2.483(1.229,5.016)	0.011*	1.977(1.085,3.602)	0.026*	1.674(0.813,3.446)	0.162	1.486(0.742,2.976)	0.263	0.443	0.055	
Now	ref	1.205(0.564,2.573)	0.631	1.448(0.750,2.796)	0.27	0.841(0.349,2.026)	0.699	1.817(0.983,3.358)	0.057	0.244	0.365	
Education												0.54
Uncollege	ref	1.579(0.887,2.809)	0.12	1.570(1.089,2.265)*	0.016	1.300(0.824,2.051)	0.259	1.437(0.977,2.113)	0.065	0.164	0.37	
College	ref	1.680(0.885,3.189)	0.113	1.537(0.826,2.858)	0.175	1.049(0.599,1.837)	0.868	1.606(0.926,2.786)	0.092	0.243	0.678	
Cancer												0.066
No	ref	1.463(0.840,2.549)	0.179	1.299(0.880,1.920)	0.188	1.068(0.667,1.710)	0.784	1.365(0.951,1.959)	0.092	0.307	0.581	
Yes	ref	1.352(0.506,3.611)	0.547	4.116(1.938,8.743)	< 0.001	1.972(0.715,5.438)	0.19	2.909(1.518,5.572)	0.001	0.001	0.837	
Race												0.64
Black	ref	0.881(0.335,2.318)	0.797	1.135(0.542,2.379)	0.737	0.956(0.449,2.035)	0.908	1.377(0.706,2.685)	0.347	0.349	0.37	
Other	ref	3.098(0.974,9.853)	0.055	1.563(0.561,4.360)	0.393	1.446(0.485,4.312)	0.508	2.505(0.854,7.354)	0.095	0.408	0.679	
White	ref	1.457(0.846,2.508)	0.175	1.773(1.185,2.653)*	0.005	1.185(0.761,1.844)	0.453	1.507(1.040,2.185)	0.03*	0.062	0.46	
Stroke												0.392
Yes	ref	3.239(1.230,8.530)	0.017*	2.560(0.920,7.124)	0.072	1.941(0.490,7.688)	0.345	1.958(0.701,5.469)	0.199	0.278	0.033	
No	ref	1.340(0.847,2.121)	0.212	1.571(1.115,2.212)*	0.01	1.121(0.752,1.671)	0.575	1.604(1.165,2.208)*	0.004	0.022	0.789	
CVD												0.443
Yes	ref	1.675(0.808,3.475)	0.166	2.444(1.346,4.438)*	0.003	1.512(0.803,2.848)	0.201	1.789(1.014,3.155)*	0.045	0.05	0.328	
No	ref	1.325(0.797,2.202)	0.278	1.365(0.850,2.192)	0.197	1.043(0.653,1.667)	0.86	1.548(1.088,2.203)*	0.015	0.09	0.702	
COPD												0.528
No	ref	1.401(0.813,2.415)	0.224	1.613(1.108,2.349)	0.013*	1.160(0.758,1.773)	0.494	1.555(1.047,2.311)*	0.029	0.071	0.874	
Yes	ref	1.950(0.894,4.251)	0.093	1.722(0.914,3.241)	0.092	1.705(0.737,3.943)	0.213	2.416(1.292,4.520)*	0.006	0.013	0.899	

## Discussion

In this prospective analysis, we examined the relationship between SUA and mortality in patients with OSA using data from the NHANES database. Our findings reveal a nonlinear association between SUA and the risk of all-cause death and cardiovascular death in the OSA population, as well as the identification of an inflection point. Both high and low levels of SUA were associated with increased risks of all-cause and cardiovascular mortality. After adjusting for demographics and disease variables, our analysis demonstrated a U-shaped curve for the association between SUA levels and the risk of all-cause and cardiovascular death, with patients having SUA levels of 5.9–6.799 mg/dl serving as the reference group. OSA patients with higher SUA levels (≥ 6.8 mg/dl) exhibited a higher risk of all-cause mortality, while those with lower SUA levels (4.4–5.2 mg/dl) had an elevated risk of both all-cause and cardiovascular mortality compared to the reference group. Given the simplicity, convenience, and cost-effectiveness of measuring SUA, this study underscores the importance of managing SUA levels to help identify high-risk groups and improve the scientific management of OSA patients' uric acid levels, ultimately reducing mortality rates.

It is worth noting that the observed all-cause mortality risk in OSA patients aligns with previous studies on the mortality risk associated with SUA levels in other populations^5, 18, 25^. Our results demonstrate a U-shaped curve, indicating that both excessively high and low uric acid levels increase the risk of all-cause mortality in OSA patients. Specifically, we found a statistically significant difference when the uric acid level exceeded 5.83 mg/dl. For every 1 mg/dl increase in uric acid levels, the risk of all-cause mortality increased by 28.6% in OSA patients with high uric acid levels. Elevated SUA levels have also been associated with increased mortality risk in patients with liver disease ^23^and DM^21^. Conversely, our results did not show a statistically significant effect on mortality in the OSA population when uric acid levels decreased below 5.83 mg/dl. Lower uric acid concentrations leading to increased mortality may be attributed to nutritional factors such as low body weight and muscle loss^26^. Considering that obesity is a major risk factor for obstructive sleep apnea^10^, it is possible that OSA patients are more prone to overnutrition rather than malnutrition, which may explain the lack of significance in patients with low-uric acid OSA. Conflicting results across studies may also be influenced by differences in cohort characteristics, sample size, and adjustment for confounders. Additionally, there is inconsistency in the reference values for SUA levels in previous studies^18^.

In general, men exhibited significantly higher levels of serum uric acid (SUA) compared to women. Previous studies have described SUA levels as an independent risk factor for mortality, irrespective of gender, while other studies have highlighted distinct disparities between men and women^16, 18, 27, 28^. Within different gender groups, a non-linear relationship between uric acid concentration and mortality risk was observed, displaying a U-shaped pattern was similar to the findings reported by Cheng^28^. Specifically, men with OSA displayed a significant inflection point at 6.219 mg/dl, whereas women exhibited a U-shaped inflection point at 5.277 mg/dl. Interestingly, there was no significant difference in uric acid concentration and mortality risk among women. However, Mazidi et al.^27^ demonstrated that SUA influenced mortality in women aged ≥ 50, whereas Zhang et al.^29^ established SUA as an independent risk factor for all-cause mortality in women of normal weight. The proportion of men and women among OSA patients differs, with men experiencing an incidence rate approximately three times higher than that of women^30^. Additionally, OSA patients generally exhibit higher uric acid levels compared to the general population^31^, with men having higher uric acid levels than women. Consequently, divergent data results between men and women among patients with OSA can be reasonably expected.

Age is another crucial factor to consider when examining the relationship between uric acid and health outcomes. Uric acid levels tend to increase with age, which can contribute to the development of age-related diseases such as gout and cardiovascular disease. Elderly individuals with high uric acid levels are more susceptible to gout and comorbidities like hypertension and DM^32^. Our analysis revealed a J-shaped curve in the relationship between SUA and death among OSA patients aged 60 and above, with an inflection point at 6.324 mg/dl. Among OSA patients aged ≥ 60 with uric acid levels below 6.32 mg/dl, each 1 mg/dl increase in uric acid was associated with a 25% decrease in mortality rate. Uric acid levels ≥ 6.32 mg/dl indicated that the increase in high uric acid had little effect on mortality. Which was similar with the study by Tseng et al.^17^, low SUA levels increased the risk of all-cause and cardiovascular mortality, potentially influenced by nutritional status. There was no statistical difference in uric acid concentration and mortality risk among OSA patients younger than 60 years old, and the underlying mechanism for this observation remains unclear and requires further investigation.

Uric acid is a complex substance that plays a significant role in the body. One theory suggests that it may promote inflammation, leading to cellular and tissue damage throughout the body and increasing the risk of chronic diseases and CVD. Another theory proposes that high uric acid levels contribute to the development of metabolic syndrome, which encompasses conditions such as obesity, hypertension, and insulin resistance, all of which are associated with increased mortality risk^33^. Numerous studies have elucidated the relationship between uric acid and mortality risk in other condition^5, 14–19^, demonstrating that both high and low uric acid levels are associated with increased mortality risk. When discussing serum uric acid, it is often associated with gout. Serum uric acid levels play a crucial role in diagnosing gout. Moreover, uric acid in urine is recognized as a cardiovascular disease (CVD) risk factor, as previous research suggests^34^. However, it is essential to acknowledge that uric acid levels can vary in different diseases or populations. The traditional cut-off values used to categorize uric acid levels as high or low are not necessarily effective in predicting disease-related mortality. Monitoring uric acid levels may serve as an important tool for predicting and preventing chronic diseases and premature death.

It is important to acknowledge the potential limitations of our study. Firstly, the diagnosis of OSA relied mainly on medical history and questionnaires, introducing potential biases, larger studies are needed to address these limitations. Secondly, the study participants were members of the United States population, so the generalizability of the results to other populations remains to be verified. Despite these limitations, our research possesses several strengths. Firstly, we employed RCS regression to explore the nonlinear relationship between SUA and OSA mortality risk, a method utilized for the first time in this context. Secondly, we conducted subgroup and sensitivity analyses for all-cause mortality data to assess effect sizes. Thirdly, we assessed death risk across the full range of SUA levels in the OSA population while considering both low and high SUA levels. Finally, in prior research, diuretics have been well-documented as factors associated with both elevated uric acid (UA) levels and increased mortality rates^35^. In this study, diuretics were included as an adjustment factor, which serves to mitigate potential biases in the results related to cardiovascular deaths.

## Conclusions

The findings of this study suggest that a U-shaped association between serum SUA levels and all-cause mortality and nonlinearity association between serum SUA levels and all-cause mortality. Further studies are warranted to determine the causal relationship between SUA level and all-cause, CVD mortality.

### Supplementary Information

Supplementary Information 1.

Supplementary Information 2.

Supplementary Information 3.

Supplementary Information 4.

Supplementary Information 5.

Supplementary Information 6.

Supplementary Information 7.

Supplementary Information 8.

## Data Availability

The datasets used and/or analysed during the current study available from the corresponding author on reasonable request.
