# Supplementary figures and images for: Association of serum uric acid with all-cause and cardiovascular mortality in obstructive sleep apnea
Source: Sci Rep. 2023 Nov 10;13:19606. doi: 10.1038/s41598-023-45508-2 (PMC10638300; doi:10.1038/s41598-023-45508-2)

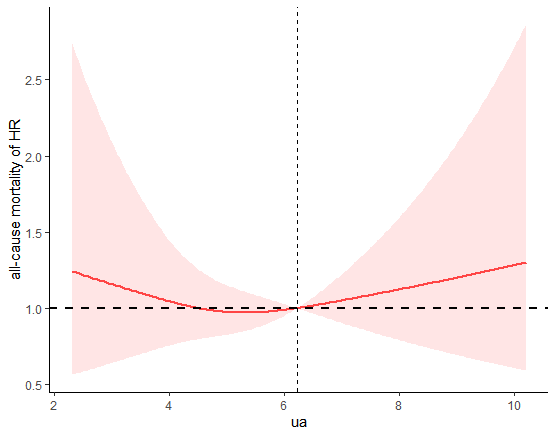

Supplement: Supplementary file 1 — Supplementary Information 1. [file 41598_2023_45508_MOESM1_ESM.tiff]

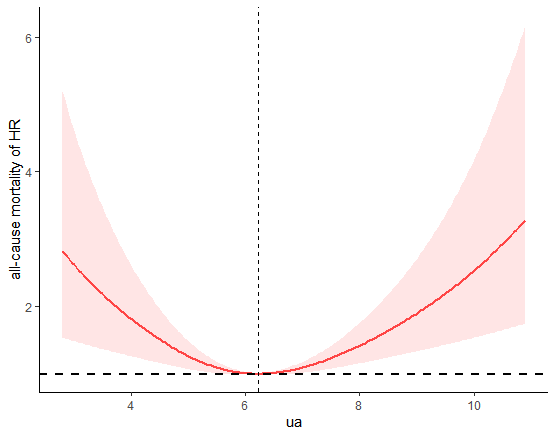

Supplement: Supplementary file 2 — Supplementary Information 2. [file 41598_2023_45508_MOESM2_ESM.tiff]

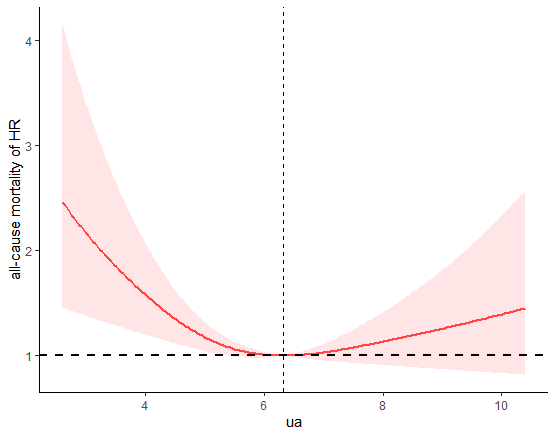

Supplement: Supplementary file 3 — Supplementary Information 3. [file 41598_2023_45508_MOESM3_ESM.tiff]

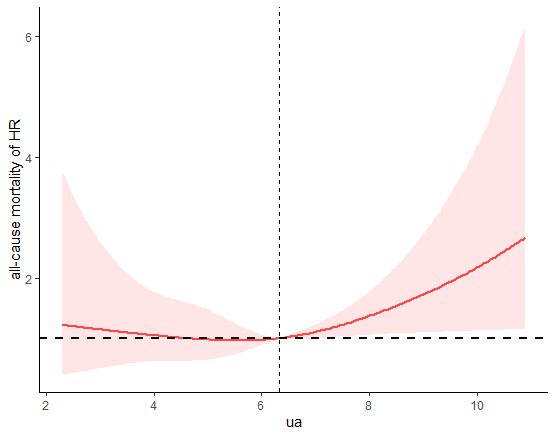

Supplement: Supplementary file 4 — Supplementary Information 4. [file 41598_2023_45508_MOESM4_ESM.tiff]
