# Supplementary material for: Association of serum uric acid with all-cause and cardiovascular mortality in obstructive sleep apnea
Source: Sci Rep. 2023 Nov 10;13:19606. doi: 10.1038/s41598-023-45508-2 (PMC10638300; doi:10.1038/s41598-023-45508-2)
Supplement: Supplementary file 5 — Supplementary Information 5. [file 41598_2023_45508_MOESM5_ESM.docx]

Supplementary figure legend

Fig S1: Multivariable-adjusted HRs for all-cause mortality by uric acid level and gender female.

Fig S2 Multivariable-adjusted HRs for all-cause mortality by uric acid level and gender male.

Fig S3 Multivariable-adjusted HRs for all-cause mortality by uric acid level and age ≥60.

Fig S4 Multivariable-adjusted HRs for all-cause mortality by uric acid level and age <60.

Table S1 Multivariable Cox regression analyses demonstrating associations of SUA and all-cause mortality by gender.

Table S2 The results of two-piecewise linear regression model between serum uric acid and all-cause mortality by gender.

Table S3 Multivariable Cox regression analyses demonstrating associations of SUA and all-cause mortality by age.
