# Supplementary material for: Association of serum uric acid with all-cause and cardiovascular mortality in obstructive sleep apnea
Source: Sci Rep. 2023 Nov 10;13:19606. doi: 10.1038/s41598-023-45508-2 (PMC10638300; doi:10.1038/s41598-023-45508-2)
Supplement: Supplementary file 6 — Supplementary Information 6. [file 41598_2023_45508_MOESM6_ESM.docx]

|  | **SUA** | | | | | |
| --- | --- | --- | --- | --- | --- | --- |
|  | Model 1 | | Model 2 | | Model 3 | |
| Male | 95%CI | P | 95%CI | P | 95%CI | P |
| Q4 | ref |  | ref |  | ref |  |
| Q1 | 2.83(1.80,4.46) | <0.0001 | 2.30(1.52,3.49) | <0.0001 | 2.00(1.11,3.59) | 0.02 |
| Q2 | 1.54(0.88,2.69) | 0.13 | 1.48(0.91,2.42) | 0.11 | 1.46(0.87,2.44) | 0.15 |
| Q3 | 1.34(0.86,2.07) | 0.19 | 1.12(0.76,1.65) | 0.58 | 1.07(0.72,1.60) | 0.73 |
| Q5 | 2.61(1.53,4.48) | <0.001 | 2.49(1.46,4.22) | <0.001 | 1.86(1.02,3.39) | 0.04 |
| p for trend |  | 0.07 |  | 0.1 |  | 0.38 |
| Female |  |  |  |  |  |  |
| Q4 | ref |  | ref |  | ref |  |
| Q1 | 0.81(0.44,1.52) | 0.52 | 0.97(0.58,1.60) | 0.89 | 0.88(0.50,1.54) | 0.66 |
| Q2 | 0.56(0.34,0.91) | 0.02 | 0.69(0.42,1.15) | 0.16 | 0.66(0.36,1.20) | 0.18 |
| Q3 | 0.60(0.32,1.12) | 0.11 | 0.74(0.43,1.27) | 0.27 | 0.69(0.41,1.15) | 0.16 |
| Q5 | 1.55(0.90,2.66) | 0.11 | 1.05(0.64,1.73) | 0.84 | 0.68(0.43,1.11) | 0.12 |
| p for trend |  | 0.26 |  | 0.99 |  | 0.08 |

TableS1 Multivariable Cox regression analyses demonstrating associations of SUA and all-cause mortality by sex.

model1: SUA

model2: SUA, age

model 3: SUA, age, DM, Hypertension, BMI, Hyperlipidemia, CVD, cancer, stroke, education, race, eGFR, CKD, smoke, COPD, HB, LYM, NEU, bilirubin, platelet count, drug of diuretic
