# Supplementary material for: Association of serum uric acid with all-cause and cardiovascular mortality in obstructive sleep apnea
Source: Sci Rep. 2023 Nov 10;13:19606. doi: 10.1038/s41598-023-45508-2 (PMC10638300; doi:10.1038/s41598-023-45508-2)
Supplement: Supplementary file 7 — Supplementary Information 7. [file 41598_2023_45508_MOESM7_ESM.docx]

| Subgroup | Adjusted HR*(95% CI) | p | p for interaction |
| --- | --- | --- | --- |
| female |  |  | 0.042* |
| inflection point(mg/dl) | 5.277 |  |  |
| <5.277 | 0.779(0.56,1.10) | 0.150 |  |
| ≥5.277 | 1.280(0.56, 1.28) | 0.448 |  |
| male |  |  | < 0.001* |
| inflection point(mg/dl) | 6.219 |  |  |
| ≥6.219 | 1.32(1.068,1.59) | 0.003* |  |
| <6.219 | 0.702(0.52,0.95) | 0.022* |  |

Table S2

The results of two-piecewise linear regression model between serum uric acid and all-cause by sex

Adjusted for the variables included demographic variables (age, sex, race, education), BMI, eGFR, RDW, HB, LYM, NEU, iron, bilirubin, platelet count, smoke, history of hypertension, DM, CKD, COPD, stroke, CVD, cancer, and hyperlipidemia, drug of diuretic.

*p<0.05
