# Supplementary material for: Association of serum uric acid with all-cause and cardiovascular mortality in obstructive sleep apnea
Source: Sci Rep. 2023 Nov 10;13:19606. doi: 10.1038/s41598-023-45508-2 (PMC10638300; doi:10.1038/s41598-023-45508-2)
Supplement: Supplementary file 8 — Supplementary Information 8. [file 41598_2023_45508_MOESM8_ESM.docx]

|  | **SUA** | | | | | |
| --- | --- | --- | --- | --- | --- | --- |
|  | Model 1 | | Model 2 | | Model 3 | |
| **Younger (<60)** | 95%CI | P | 95%CI | P | 95%CI | P |
| Q4 | ref |  | ref |  | ref |  |
| Q1 | 0.72(0.31,1.69) | 0.45 | 0.91(0.31,2.65) | 0.86 | 0.84(0.25,2.80) | 0.78 |
| Q2 | 0.79(0.42,1.47) | 0.45 | 0.90(0.44,1.82) | 0.77 | 0.89(0.46,1.74) | 0.74 |
| Q3 | 0.65(0.33,1.29) | 0.22 | 0.68(0.34,1.34) | 0.26 | 0.68(0.33,1.41) | 0.30 |
| Q5 | 1.29(0.76,2.19) | 0.34 | 1.26(0.80,1.99) | 0.33 | 1.17(0.77,1.75) | 0.46 |
| p for trend |  | 0.43 |  | 0.68 |  | 0.82 |
| Elder (≥60) |  |  |  |  |  |  |
| Q4 | ref |  | ref |  | ref |  |
| Q1 | 1.60(1.04,2.45) | 0.03 | 1.64(1.10,2.46) | 0.02 | 1.92(1.20,3.09) | 0.01 |
| Q2 | 1.62(1.13,2.34) | 0.01 | 1.76(1.25,2.49) | 0.001 | 1.89(1.29,2.76) | 0.001 |
| Q3 | 1.13(0.75,1.71) | 0.56 | 1.28(0.86,1.91) | 0.22 | 1.46(0.94,2.28) | 0.10 |
| Q5 | 2.25(1.45,3.49) | <0.001 | 2.27(1.46,3.52) | <0.001 | 1.81(1.07,3.05) | 0.03 |
| p for trend |  | 0.004 |  | 0.004 |  | 0.07 |

TableS3 Multivariable Cox regression analyses demonstrating associations of SUA and all-cause mortality by age.

model1: SUA

model2: SUA, sex

model 3: SUA, sex DM, Hypertension, BMI, Hyperlipidemia, CVD, cancer, stroke, education status, race, eGFR, CKD, smoke, COPD, HB, LYM, NEU, bilirubin, platelet count, drug of diuretic
